# Self-Sensing of a Magnetically Actuated Prism

**DOI:** 10.3390/s23125493

**Published:** 2023-06-10

**Authors:** Pascal M. Weber, Ulrike Wallrabe, Matthias C. Wapler

**Affiliations:** 1 Laboratory for Microactuators, Department of Microsystems Engineering (IMTEK), University of Freiburg, 79085 Freiburg, Germany; wallrabe@imtek.uni-freiburg.de; 2 Laboratory for Microsystems Engineering for Medical Engineering, Faculty of Electrical Engineering and Information Technology, Otto-von-Guericke University Magdeburg, 39016 Magdeburg, Germany; matthias.wapler@ovgu.de

**Keywords:** self-sensing, coil impedance measurement, magnetic actuation, Bi-axial tunable prism

## Abstract

We demonstrate a method for self-sensing of a magnetically actuated prism that can be used, e.g., in a feedback-loop without the need of additional sensors. In order to use the impedance of the actuation coils as a measurement parameter, we first obtained the optimal measurement frequency that is well separated from the actuation frequencies and at the same time provides the best compromise between sensitivity to the position and robustness. We then developed a combined actuation and measurement driver, and correlated its output signal to the mechanical state of the prism using a defined calibration sequence. We demonstrate that we can reliably measure the state of each actuator and determine the tilt angle of the prism with an accuracy of ±0.1${}^{\circ }$ in the polar angle over a range of ±4${}^{\circ }$ and ±20 mrad in the azimuthal angle.

## 1. Introduction

Adaptive optics offer a great way to improve the performance of an optical system while reducing its complexity at the same time. For example, galvo or MEMS mirrors are commonly used for scanning applications. However, they have the disadvantage that they fold the beam path, resulting in a bulkier setup and requiring a precise alignment as each mirror doubles the alignment error. To avoid this issue, we have developed piezo electrically [1] and magnetically [2] actuated transmissive prisms for bi-axial beam steering and have already successfully used them in a microscope setup [3].

Like with most systems, the performance of the optical setup can be enhanced with a suitable closed loop control. In optics, this can be performed by measuring the wavefront with a Shack–Hartmann sensor after the actuated element [4]. Though there have been efforts to combine the actuated and measuring device [5], they still remain separated either physically or in the time domain. Another approach is to measure the state of the actuated device, which can be used for galvo [6] and MEMS [7] mirrors. However, this requires additional sensors, increasing the complexity and size of the device.

As the magnetically actuated prism still suffers from hysteresis because of the elastomers in the support structures [2,8], the final step is the realization of a closed loop control. For example, to provide an accurate mapping when performing scanning microscopy. To avoid the drawbacks of additional sensors or hardware on the device, we propose the use of self-sensing for the prisms’actuators to determine the state of the prism, using the example of the pure prism. In general, self-sensing utilizes the fact that most actuators can, to a certain degree, be operated in both directions, and also as a sensor. This means that the state of the device influences the properties of the actuators which can be observed to gain information of the state of the system [9]. Usually, a complex system model is needed to separate the influence of the state of the system and its control input [10,11]. As such information is not always available, we are going to present a sensing method that is not affected by the driving signal. To achieve this, we will characterize the actuation coils for different states of the prism and determine which properties can be used for self-sensing in Section 2. In Section 3, we present a circuit that measures those properties and translates changes into a DC output voltage. Finally, in Section 4 we will relate those output signals to the state of the prism and measure the accuracy of this method.

## 2. Magnetically Actuated Prism

Before characterizing the prism actuation coils, let us take a brief look at the design and the actuation method of the prism.

### 2.1. Mechanical Setup

**Figure 1:**
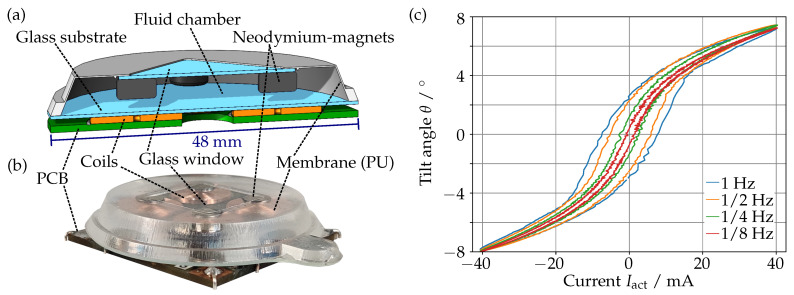
Cross-section (**a**) [2] of the magnetically actuated prism (**b**). Tilt angle [2] of the glass window for a sinusoidal current with different frequencies (**c**).

Figure 1a shows a cross-section through the magnetically actuated prism (b) as we have presented it in [2]. The optical path consists of a fixed glass substrate at the bottom and a movable glass window at the top. The glass windows are held in place by a 200 μm thin polyurethane membrane that connects and seals the glass components. The resulting fluid chamber is filled with paraffin oil, forming the optical body of the prism. To actuate the prism, we bonded four neodymium magnets to the corners of the glass window that can be pushed up or pulled down by their corresponding coil under the glass substrate. By actuating each coil individually with an actuation current $I_{\mathrm{act}}$, it is possible to tilt the glass window in every direction and to displace it vertically in a piston movement. The coils are not bonded to the glass substrate, but to a PCB that is held in place by the housing of the prism. This allows for an economical fabrication and straightforward exchange of the passive optical part or the active actuation coils. This configuration, in particular the use of robust and soft materials, makes the prism tolerant to physical mishandling.

However, as can be seen in Figure 1c, the prism has a nonlinear mechanical response, with a significant frequency-dependent hysteresis. Furthermore, the freely floating glass window makes the prism sensitive to gravitational effects when tilting it sideways. To counteract these effects and to increase the long-term reliability, we need a method of measuring the position of the glass window. This, of course, could be achieved straightforwardly with additional sensors on the device, resulting in a more complex mechanical design. To keep the complexity of the device at a minimum, we will demonstrate a self-sensing approach, where each actuation coil actuates the prism while measuring the distance to the magnet above at the same time.

### 2.2. Frequency Spectrum of the Actuation Coils

As the impedance can be used to describe all parameters of a coil, it will reflect any change in the interactions with the coil and its environment.

Using a phenomenological approach, the details of how the change in impedance is caused are not relevant, in particular, as the different effects, such as the remaining permeability of the neodymium magnets, the self-inductance in the magnets and the mechanical interaction with the prism cannot be clearly separated. The aim of this section is hence to verify that we indeed see a significant sensitivity of the impedance of the actuation coils to the mechanical state of the prism and to find a measurement frequency $f_{\mathrm{meas}}$, where the impedance is most sensitive and reliable.

For this, we measured the impedance spectrum over a wide frequency range for different static actuation states, i.e., for different actuation currents, of the prism. To avoid any electrical interaction between the driving current and the measurement, we used the symmetry of the prism and actuated the coil at the opposite end of the prism when measuring a given coil; if one magnet is pulled down, the magnet on the other side of the glass window will move up, and vice-versa. A positive current gives a smaller distance between the measured coil and its magnet, while a negative current increases the distance.

**Figure 2:**
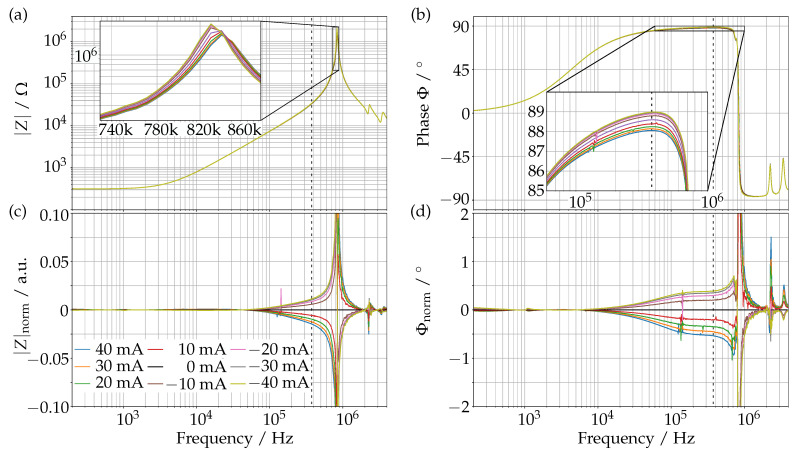
Absolute value (**a**) and phase (**b**) of the impedance *Z* of the actuation coils as functions of the frequency for different actuation states described in the text. For a better comparison, see the change in the impedance normalized to the unactuated measurement (**c**), and the phase difference to the unactuated measurement (**d**).

Figure 2 shows the impedance spectrum with the absolute value (a) and the phase (b). We see the typical behavior of an inductor. For low frequencies it approaches a dc resistance of 316 Ω, and we have the resonance due to the parasitic capacitance at around 836 kHz. The two insets show that there is indeed a change in the impedance, notably a small shift of the resonance frequency and a change in the maximum phase angle. To obtain a better view of these changes, we plotted the absolute value, normalized to the unactuated measurement (0 mA), ${\left|Z\right|}_{\mathrm{norm}}$ (c) and the phase difference to the unactuated measurement (d). It can be seen that the differences in ${\left|Z\right|}_{\mathrm{norm}}$ increase as we approach the resonance frequency. However, picking it as measurement frequency $f_{\mathrm{meas}}$ may not be optimal as even a small deviation of $f_{\mathrm{meas}}$ gives a large change and can even invert the sensitivity. Hence, we choose a frequency of $f_{\mathrm{meas}}=376\;\mathrm{kHz}$, which is indicated with a dotted line in the plots.

At this frequency, the change in ${\left|Z\right|}_{\mathrm{meas}}$ is sufficiently large, while the phase has a nice plateau, making the system more tolerant to small deviations of $f_{\mathrm{meas}}$. This frequency is also well above any possible actuation frequencies that are well below 1 kHz [2].

### 2.3. Impedance–Displacement Mapping

After having $f_{\mathrm{meas}}$ determined, it is now possible to measure the impedance for a dynamic actuation of the prism. We used again the symmetry of the device by actuating three of the coils while measuring the fourth one. This is performed by using a sinusoidal current with an amplitude of 40 mA and a period of 8 s. Simultaneously, we used a confocal distance sensor to measure the displacement *h* of the magnet above the measured coil.

**Figure 3:**
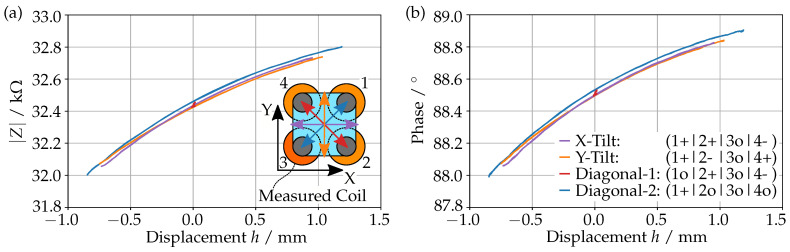
Absolute value (**a**) and phase (**b**) of the impedance as a function of the displacement *h*, for different actuation directions. The inset show the top view of the prism in the coordinate system. The actuation directions are displayed as arrows in the corresponding colors.

The displacement-dependent impedance can be seen in Figure 3, where we see the absolute value (a) and the phase (b) for different actuation directions. The inset shows the top view of the prism with its four coils in the coordinate system. The direction of the actuation is shown as arrows in the corresponding color. To tilt the glass window of the prism in the Diagonal-1 direction (red) we actuated coil 2 and coil 4 in opposing directions, while coil 1 and 3 remained neutral (1o|2+|3o|4-). For the X-Tilt (purple) we actuated coil 1 and 2 opposing to coil 4 (1+|2+|3o|4-), and similarly, coil 1 and 4 opposing coil 2 for the Y-Tilt (orange). In all cases coil 3 remained unactuated as it was used for the impedance measurement. This includes Diagonal-2 (blue) where only coil 1 was actuated (1+|2o|3o|4o).

It can be seen that the phase and absolute value depend primarily on the displacement, as the data for different actuation directions are very close to each other. This observation is further strengthened by the Diagonal-1 direction. Here the displacement stays constant, but the magnet tilts with the glass window in front of the coil. As can be seen in (a) and (b), neither the phase nor the absolute value react to such changes. In particular, we also do not see any hysteresis in this plot.

## 3. Method

In the previous section, we have verified that the impedance of the actuation coil relates to the distance to the magnet above. In the following, we will present the technique to measure this very small change in impedance.

### 3.1. Measurement Principle

**Figure 4:**
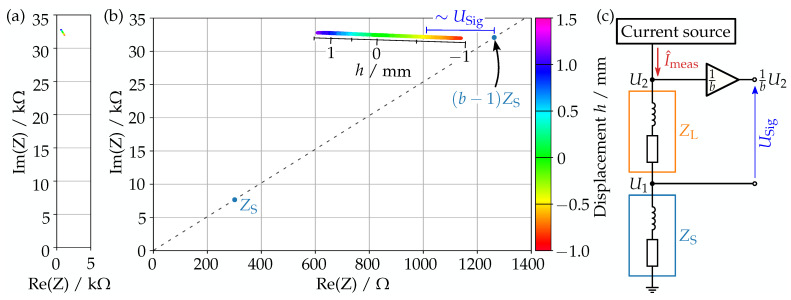
Impedance of the actuation coil in complex space for actuations in different directions, with the displacement *h* given by colors and indicated by the additional axis. Uniform axis scaling (**a**) or magnified (**b**). Here we see also the reference point $\left(b-1\right)Z_{S}$ as it is created with the measurement setup (**c**).

Figure 4a,b shows the variation of the impedance in complex space for different actuation directions, with the displacement *h* of the magnet coded in color. As can be seen in (b), all measurement points align to one line, sorted by *h*. The results look also identical at different actuation frequencies. This, together, shows that the impedance indeed depends only on the displacement.

Looking at the data with uniform axis scaling in Figure 4a, we see, however, that the total change in impedance is very small. This means we require a highly precise measurement.

Our approach is hence to measure the impedance with respect to an appropriate reference point in complex space. This can be performed using the small shunt impedance $Z_{S}$, which is connected in series with the actuation coil $Z_{L}$ as we can see in Figure 4c and which is normally used for the feedback of the current source. We can use this to create the reference point $\left(b-1\right)Z_{S}$ by dividing $U_{2}$ by a fixed value *b* and then measuring the difference to $U_{1}$ as illustrated in Figure 4c. The resulting signal $U_{\mathrm{Sig}}$ is then given by

(1) $$U_{\mathrm{Sig}}=\frac{1}{b}U_{2}-U_{1}.$$

Under the assumption that the same measurement current $I_{\mathrm{meas}}$ flows through $Z_{L}$ and $Z_{S}$, this becomes

(2) USig=Imeas1b︸Term1ZL−b−1ZS1b︸Term2.

$U_{\mathrm{Sig}}$ is then proportional to the difference between $Z_{L}$ and $\left(b-1\right)Z_{S}$ as we illustrate also in Figure 4b. The value for *b* can be determined either analytically from $Z_{S}$ and $Z_{L}$ or experimentally by measuring $U_{1}$ and $U_{2}$ for different displacements of the magnets to calculate the output behavior for different *b* values, choosing the one with the best sensitivity.

### 3.2. Measurement Circuit

**Figure 5:**
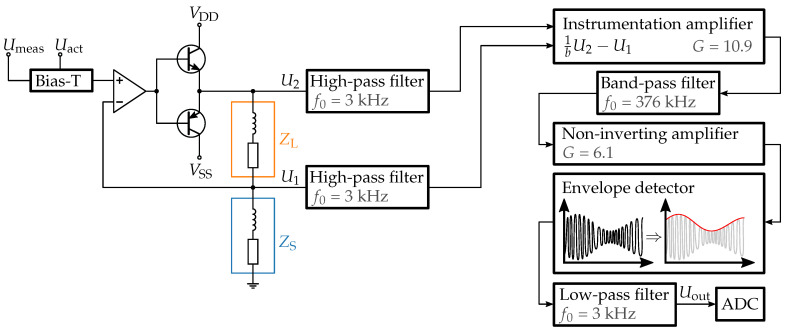
Block diagram of the measurement circuit. The measurement and actuation signal are mixed with a Bias-T before being converted into a current. High-pass filters are used to separate the measurement signal again before it is further processed.

To realize the measurement principle, we developed the circuit shown in Figure 5. In the beginning, the high-frequency measurement signal and the low-frequency actuation signal are mixed using a Bias-T. The current source consists of an op amp (*LT1360*) in combination with two bipolar transistors (NPN: *2SC5706*, PNP: *2SA2039*) and the shunt impedance $Z_{S}$. To avoid saturation effects that add significant complexity [12] we used air coils for both the actuation coil and $Z_{S}$.

Because $U_{1}$ and $U_{2}$ depend on the measurement and actuation signals, we used two high-pass filters to suppress the actuation signal. This is performed with a 1 GΩ impedance buffer (*OPA1612*) and a second-order RC filter with a cutoff frequency of about 3 kHz. In the next function block, Equation (1) is realized by dividing $U_{2}$ with a simple voltage divider and then measuring the difference to $U_{1}$ using an instrumentation amplifier (*AD8421*). Based on the impedance $Z_{S}$ of our shunt coil, we choose a value of b=6 to place the reference point according to Figure 4b, and set the amplification of the instrumentation amplifier to G=10.9.

Afterwards, the signal of the instrumentation amplifier passed through a passive RC band-pass filter with a center frequency corresponding to the measurement frequency of $f_{\mathrm{meas}}=376\;\mathrm{kHz}$ to suppress any interference signal. To obtain a low-frequency signal that directly relates to the mechanical state of the prism, we finally insert a simple envelope detector using a *1N4148* diode. To overcome the threshold voltage of the diode, we first pass the signal through a non-inverting amplifier with an amplification of G=6.1. After the envelope detector, the signal is passed through an additional 3 kHz low-pass filter before being measured with an analog-to-digital converter.

## 4. Characterization

To characterize the measurement circuit, we measured its input–output behavior, tested to which degree it is susceptible to the actuation current and compared the actual tilt angle to the one determined with the measurement circuit.

### 4.1. Input–Output Behaviour

**Figure 6:**
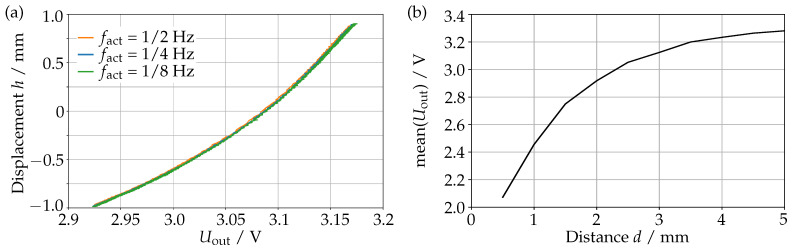
Measured displacement *h* of the of the magnet as a function of the output voltage $U_{\mathrm{out}}$ from the measuring circuit, for different actuation frequencies (**a**). Mean of $U_{\mathrm{out}}$ when sweeping $I_{\mathrm{act}}$ on a fixed set of distances *d* between the coil and a fixed magnet (**b**). Error bars indicating the variation over different actuation currents would not be visible.

To measure the input-output behavior we used a similar setup as in Section 2.3. Now, however, our measurement circuit is used to drive and measure the actuation coils at the same time. The resulting data can be seen in Figure 6a, where we see the measured displacement *h* of the magnet as a function of the output signal $U_{\mathrm{out}}$ from the measurement circuit of the corresponding coil.

For the actuation we choose a Diagonal-2 direction because it gives the largest displacement, and we had to lower the amplitude of the actuation current to 36 mA in order not to exceed the driving range of the current source. As a first indication for the reliability, we measured this for three different frequencies as shown in the figure. We can see that the input–output behavior does not depend significantly on the frequency. Furthermore and most importantly, we can see that none of the measurement data show a hysteresis.

To test whether the actuation current influences the output signal $U_{\mathrm{out}}$, we replaced the prism above the coils with a magnet that is held firmly in place by a polyamide screw, at a position precisely determined by FR2 phenolic paper shims between the coil and the magnet. We then measured $U_{\mathrm{out}}$ for different distances *d* between coil and magnet while applying several dynamic and static actuation currents. The RMS variation of $U_{\mathrm{out}}$ for different actuation currents did not exceed 5.5 mV, allowing the conclusion that the actuation current does not effect $U_{\mathrm{out}}$. The mean of $U_{\mathrm{out}}$ can be seen in Figure 6b as a function of *d*; error bars indicating this RMS variation would not be visible. We see that the sensitivity of the device decreases with increasing *d*. Note that this distance *d* and the displacement *h* are correlated but not the same, as *d* describes the physical distance between coil and magnet and *h* the displacement from the idle state of the prism measured at the approximate center point of the magnet.

### 4.2. Measurement of the Tilt Angle

**Figure 7:**
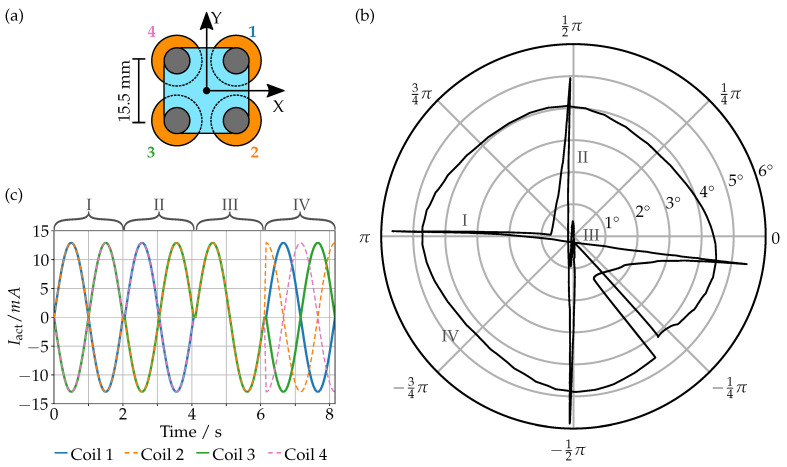
Top view of the prism in the coordinate system and the assigned number for each coil (**a**). Measured tilt angle (polar) and direction (azimuthal) of the prism when being actuated with the calibration sequence (**b**). Current curve for each coil that is used as a calibration sequence (**c**).

As we have shown in the previous section, we can reliably measure the vertical position of each individual magnet. This can be used to determine the tilt angles of the prism and the vertical offset. To determine the position, we first parametrize the position of each magnet as a function of the corresponding $U_{\mathrm{out}}$ using a simple polynomial fit of the third degree. Then, we obtain the X- and Y-tilt and the offset from the combination of the displacements of three of the magnets and the effective positions where the displacements are measured. To determine those effective positions and the polynomial fits, we actuated the prism with a current curve as can be seen in Figure 7c.

The calibration sequence combines a tilt around the X-axis (I), the Y-axis (II), a piston (III) and a circle (IV) movement. The corresponding trajectory of the tilt angles can be seen in Figure 7b, measured by scanning the glass window pointwise with a confocal distance sensor. The output signal $U_{\mathrm{out}}$ for each coil was captured simultaneously, allowing us to fit the coefficients and measuring positions as shown in Table 1 by fitting. When comparing the positions with their actual separation (15.5 mm), we notice that the effective measurement position is not necessarily the center point of each coil but they are in a reasonable order of magnitude.

**Figure 8:**
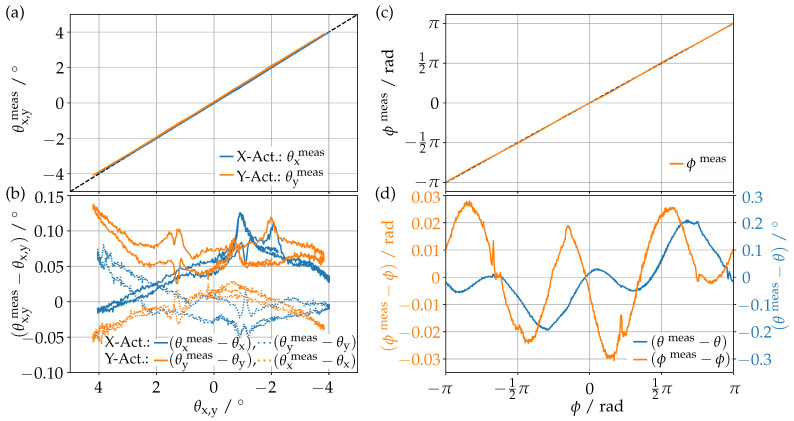
Tilt angles for X and Y obtained from the self-sensing method, $\theta_{x,y}^{\mathrm{meas}}$ as a function of the actual tilt angle $\theta_{x,y}$, when the prism is actuated along the corresponding axis (**a**). Difference between measured and actual tilt angle for both actuations and their cross-talk (dotted lines) (**b**). Measured azimuthal angle $\phi^{\mathrm{meas}}$ as a function of the actual azimuthal angle ϕ when the prism is actuated in a circle (**c**). Difference between measured and actual azimuthal and polar angle θ (**d**).

With those parameters, it is now possible to obtain the tilt angles of the glass window from the outputs of the measurement circuits. To characterize our self-sensing method, we compare its result with a precise measurement from the confocal distance sensor. This is performed for three different actuations, a tilt in the X and Y directions (Figure 8a,b) and a circular movement (Figure 8c,d). Figure 8b, furthermore, shows that we are also capable of detecting the cross-talk, i.e., the deflection in the neutral direction. To keep the coils and circuitry at an approximately constant temperature, we used sinusoidal currents of the same amplitude and frequency as in the calibration sequence.

In Figure 8a, we show the measured tilt angle $\theta^{\mathrm{meas}}$ as a function of the actual angle θ for the actuations in the X and Y directions. As can be seen, both actuations are very close to the identity function, i.e., the self-sensing sensors are well suited to measure the tilt angles precisely. This can also be seen in Figure 8b, where the difference between ideal and measured angle shows a deviation of less than 0.15${}^{\circ }$ with an RMS of 0.03${}^{\circ }$.

Similarly, Figure 8c shows the measured azimuthal angle $\Phi^{\mathrm{meas}}$ from the circular actuation. This confirms that the azimuthal angle can also be measured reliably, as the deviations in Figure 8d do not exceed 30 mrad for the azimuthal and 0.2${}^{\circ }$ for the polar angle, with an RMS of 20 mrad for the azimuthal and 0.1${}^{\circ }$ for the polar angle. Still, those deviations seem systematic, indicating a limitation of our method, but also an opportunity to correct the remaining inaccuracy.

## 5. Summary and Conclusions

We successfully implemented the concept of self-sensing for a magnetically actuated prism using the impedance of the actuation coils at a fixed measurement frequency $f_{\mathrm{meas}}$. By measuring the impedance spectrum of the actuation coil for a set of different actuation states, we first determined $f_{\mathrm{meas}}=376\;\mathrm{kHz}$, where we found the best correlation between the impedance and the state of prism, well above the speed of the mechanical response. To build a circuit that can drive and measure an actuation coil at the same time, we combined the high measurement frequency with a slow actuation signal using a Bias-T and extracted the measurement signal using appropriate filters. The sensing part of the circuit is completed by measuring the difference between the impedance of the actuation coils and a reference impedance in complex space. We then measured a defined calibration sequence consisting of tilts along the primary axis, a circle and a vertical displacement movement to determine the virtual position of the displacement measurement corresponding to each coil in addition to the polynomial coefficients that relate the output voltage to the displacement.

With these parameters, we have shown that we can measure the tilt angles of the prism using the feedback of three of the four actuation coils. We achieved an RMS accuracy of ±0.1${}^{\circ }$ (max. 0.2${}^{\circ }$) for the polar angle over a sensing range of ±4${}^{\circ }$, and an error below 30 mrad with an RMS accuracy of ±20 mrad in the azimuthal angle.

While we worked at a constant temperature, the temperature of the coils could be monitored using its DC resistance. By sensing all four actuators of the prism, it should be possible to implement a parameter estimation, allowing the device to adapt to temperature changes and to improve the long-term stability. With this being implemented it should be feasible to realize a suitable closed loop control for the prism without the need of additional sensors.

## Figures and Tables

**Table 1:** Fitted X and Y positions where each of the four coils measure the displacement of its magnet above.

Axis	Coil 1	Coil 2	Coil 3	Coil 4
X/mm	6.78	6.71	−6.90	−6.09
Y/mm	7.04	−5.83	−6.33	6.52

## Data Availability

All relevant data are contained within the document.
